# Frame Score, Grazing and Delayed Feedlot Entry Effect on Performance and Economics of Beef Steers from Small- and Large-Framed Cows in an Integrated Crop-Livestock System

**DOI:** 10.3390/ani11113270

**Published:** 2021-11-15

**Authors:** Songul Şentürklü, Douglas Landblom, Steven Paisley, Cheryl Wachenheim, Robert Maddock

**Affiliations:** 1 Dickinson Research Extension Center, North Dakota State University, Dickinson, ND 58601, USA; 2 Department of Animal Science, Canakkale Onsekiz Mart Universitesi, BMYO, Canakkale 17200, Turkey; 3 Department of Animal Science, University of Wyoming, Laramie, WY 82071, USA; SPaisley@uwyo.edu; 4 Department of Agribusiness and Applied Economics, North Dakota State University, Fargo, ND 58105, USA; cheryl.wachenheim@ndsu.edu; 5 Animal Science Department, North Dakota State University, Fargo, ND 58105, USA; robert.maddock@ndsu.edu

**Keywords:** annual forage, cow size, delayed feedlot entry, grazing economics, perennial-annual forage, retained ownership, steer frame score

## Abstract

**Simple Summary:**

Small-framed cows require less pasture forage per animal unit compared to their larger-framed counterparts. Small-framed cows whose mature size has been reduced by breeding moderate to large-framed cows to small-framed Aberdeen Angus (Lowline) sires produce progeny that are often subject to marketing bias within the USA marketing system, when they are sold at weaning as feeder cattle destined for feedlots (approximately seven months of age), because their post-weaning growth and efficiencies are less. In lieu of selling small-framed steers at weaning, delaying feedlot entry by retaining ownership of progeny through the wintering period followed by grazing a sequence of perennial and annual forages grown on marginal semi-arid cropland for 212 days before feedlot finishing is a management protocol that eliminates market bias. Extended grazing prepares small-frame steers for compensatory gain during feedlot finishing, reduces feedlot feeding days by 62.4%, improves growth, supports comparable feed efficiency, reduces finishing feed cost, reduces finishing feed cost per kg of gain, yields exceptional meat quality (97% Choice quality grade), and increases net return to the integrated system.

**Abstract:**

When selling small-framed steers at weaning, profitability is diminished. The hypothesis is that by using a vertically integrated business model that includes retained ownership, extended grazing, abbreviated feedlot finishing, and selling at slaughter, profitability would increase. Crossbred yearling steers (*n* = 288) from small size Aberdeen Angus (Lowline) × Red Angus × Angus × Angus cows and moderate to large size Red Angus × Angus × Simmental × Gelbvieh cows calved May−June were randomly assigned (complete randomized design), in a 3 y study, to feedlot control (FLT) and extended grazing (GRZ) frame score treatment groups. Mean frame score for FLT were small frame (SF) 3.82 and large frame (LF) 5.63, and for GRZ, SF: 3.77 and LF: 5.53. Least-square means were utilized to identify levels of effects and to control family-wise error adjusted with Tukey test. The FLT control steers were housed in the feedlot and fed growing diets and subsequently high energy corn-based diets for 218 days. The GRZ steers grazed a sequence of forages (native range, field pea-barley mix, and unharvested corn) for 212 days and then were transferred to the feedlot and fed high energy corn-based finishing diets for 82 days. The SF GRZ steers grew more slowly grazing native range and annual forages compared to GRZ LF steers, but SF steer grazing cost per kg of gain was reduced 7.80%. Grazing steers did not grow to their full genetic potential. Slower growth during grazing allowed LF and SF steers to grow structurally before feedlot entry creating a compensatory feedlot finishing growth response. Overall, grazing steer performance exceeded steer performance of the FLT control treatment and LF grazing steers had the highest rate of gain, and lowest feed cost per kg of gain. The GRZ steer feedlot days on feed were reduced 136 days and total feed intake was reduced resulting in LF and SF grazing steer feed cost reductions of 175.9 and 165.3%, respectively. Extended grazing also resulted in LF and SF grazing steer hot carcass weights to be greater than control LF and SF steers and SF grazing steers had greater dressing percent, and marbling score. Carcass quality grade, meat tenderness, and cooking losses were similar. System net returns were highest for LF (USD 911.58), and SF (USD 866.61) grazing steers. Managerial modification combining retained ownership, extended grazing, and delayed feedlot entry increased profitability and eliminated market bias.

## 1. Introduction

Worldwide, controlling the costs and capturing profitability are challenging and often influenced by factors completely out of the cattle producer’s control. Risk can be mitigated in part by creating greater net beef value before the first point of sale. One approach is retained ownership within a vertically integrated system from birth to slaughter and secondly, reducing cow size.

Brood cow size has a direct effect on business input costs and interacts with the cow’s biological efficiency, which is a complex interaction accounting for reproduction, available feed resources, frame size, milking ability, and growth [1,2]. When feed resources are limited in semi-arid environments, smaller cows with lower milk production are more efficient, whereas larger cows are unable to maximize their full genetic potential [3]. Matching cow size to the environment requires an assessment of the environmental conditions and a disciplined approach to meet the needed cow size nutrient requirements. In semi-arid environments, slaughter cow carcass weight has increased over time from 227 kg in 1960 to 290 kg in 2020 and during the same timeframe, steer carcass weights have increased from 295 to 408 kg [4]. Growth characteristics and expected progeny difference selection tools have contributed to increasing cow size and progeny finished weight [5,6]. These selection tools can also be employed to reduce cow size when environmental and other conditions warrant it. Reducing cow size is a management technique that ranchers can use to control costs and increase profitability. Efforts for changing cow size can occur slowly over time using multiple selection over generations, or rapidly mating bulls from smaller frame-size cattle breeds (3.5–4.0) to cows of larger frame size (5.5–6.5).

Once cows are genetically transformed to a smaller frame score, there is a greater potential for market bias when compared to calves from moderate to large frame size cows. Marketing of progeny from herds with differing cow size can be problematic due to discounts for small frame steers and heifers and, therefore, investigations into management alternatives is warranted.

Crop production systems in the semi-arid regions of the USA, where a wide variety of adapted crops can be grown in diverse crop rotations, hold promise for increasing profitability from small frame-size calves. Yearling systems that utilized native range (NR) and annual forage (ANN) grazing within a diverse, multicrop, 5-year rotation had an enhanced, economically important grazing growth, muscle, marbling, and profitability following extended grazing and delayed feedlot entry, when compared to a traditional feedlot growing and finishing program [7]. Alternatively, managerial adjustments that include extended grazing options give the cattle producer greater control over inputs with reduced risk, and increased profit potential. The feeding of harvested feeds instead of grazing increases slaughter breakeven costs [8], whereas extensive, managed cattle grazing followed by an abbreviated concentrate-feeding period has more upside profitability potential [9]. In lieu of marketing calves directly after weaning, retaining ownership coupled with extended summer grazing allows producers to capitalize on compensatory growth [10], reduced slaughter closeout cost [11], and expand the integrated system net profit [12]. The objective of this study was to evaluate yearling steer progeny produced from mating small frame-size Aberdeen Angus (Lowline) bulls to Angus cows with 5.5–6.0 frame size, and within a retained ownership business model, compare a traditional feedlot growing−finishing system to a long-term extensive grazing system, with reduced feedlot residency, and document grazing and feedlot performance, carcass characteristics, meat tenderness and cooking losses, and systems economics among yearling steer progeny from small- and large-frame cow matings.

The research hypothesis was that small-frame steers managed extensively grazing a sequence of native range and annual forages for more than 200 days followed by an abbreviated finishing period of less than 90–100 days would be profitable and eliminate marketing bias.

## 2. Materials and Methods

### 2.1. Ethics Statement

The North Dakota State University Institutional Animal Care and Use Committee (protocol #A12007) reviewed and approved protocols for animal use in this investigation.

### 2.2. Research Site and Environmental Conditions

Native range and ANN forage grazing components of this research were conducted at the Dickinson Research Extension Center ranch headquarters (14°11′40″ N latitude, 102°50′23″ W longitude) located 35 km north of Dickinson, ND, USA. The region is semi-arid with an average long-term precipitation from April to October of 311.9 mm and average maximum and minimum temperatures of 23.8 °C and 8.5 °C [13].

### 2.3. Animals and Grazing Treatments

Two hundred and eighty-eight November-weaned steer calves born during May−June each year, of a three-year study, were grown during the winter (November to April) as a common group, after a 7-day drylot weaning-recovery period, grazing unharvested corn (UCN; Z. maize), corn residue, and supplemental medium quality alfalfa-bromegrass mixed hay (*Medicago sativa* and *Bromus inermis*) plus 0.91 kg/day of a 32% CP distiller’s dried grain-based supplement.

The steers that averaged 12.0 months of age were randomly assigned based on weight, age, and frame score to small frame (SF) and large frame (LF) feedlot control (FLT) groups and SF and LF extended grazing (GRZ) treatment groups. Within treatment, frame score mean values for the FLT control were SF: 3.82 and LF: 5.63, and for GRZ, mean values were SF: 3.77 and LF: 5.53. Steer frame score values were determined at November weaning. Using 205-day hip height and weight, steer frame score was computed according to Beef Improvement Federation Guidelines [14]: −11.548 + (0.4878 × Ht) − (0.0289 × Age) + (0.00001947 × Age2) + (0.0000334 × Ht × Age), where height (Ht) is measured in inches and age in days. The steers originated from Aberdeen/Lowline (LO × AR × AN × AN) and traditional (AR × AN × SM × GV) crossbred cowherds maintained at the Dickinson Research Extension Center. Each year of the three-year study, control treatment SF and LF steers were randomly assigned to three feedlot pen replicates per frame score group (*n* = 8 steers per feedlot pen replicate; 24 steers per frame score group; FLT treatment total: *n* = 48). For the grazing treatment, the SF and LF grazing groups grazed triple replicated 1.74 ha annual forage fields (*n* = 8 steers per forage field replicate; 24 steers total per frame score group; GRZ treatment total: *n* = 48).

### 2.4. Diets, Forage and Feeding System

The first week of May each year, SF and LF steers allotted to the FLT control treatment were transferred directly to the University of Wyoming, Sustainable Agriculture Research Extension Center feedlot, Lingle, WY, USA for growing and finishing. Steers were housed in pens (33.5 m long × 6.1 m wide × 1.52 m high) constructed of continuous high-tensile, five-wire, electric fence (3 wires energized/2 ground: Gallagher XL 25 Joule System, Gallagher USA Electric Fence, Riverside, MO, USA), continuous concrete feed bunks, neck cable fixed at 40.6 cm above bunk edge, and continuous flow waterers. Steers received calf-hood vaccinations at two months of age and a booster vaccination at seven months of age using Ultrabac 8 and Bovi-Shield Gold One Shot (Zoetis, Parsippany NJ, USA). Hormone growth implants were not used in this study and horn and face flies were controlled with monthly releases of parasitic wasps (Kunafin, Quemado, TX, USA). In the feedlot, steer health and bunk readings were performed at 0730 h each morning and recorded. Total mixed rations were delivered to pens twice daily at 0800 h and 1430 h. The total mixed ration dry matter formulation, ingredients, and nutrient analysis are shown in Table 1.

The diet formulation consisted of alfalfa haylage (*Medicago sativa*, 6 h wilt), alfalfa hay (*Medicago sativa*), wheat straw (*Triticum aestivum*), whole corn (*Zea mays, indentata*), and a medicated feedlot vitamin-mineral. Diet energy increased seven times over 135 d from the initial receiving diet (69.6% TDN; NEg 193.0 MJ/45.4 kg) to the final finishing diet (84.0% TDN; 265.4 MJ/45.4 kg) and included 250 mg Rumensin/steer/day (monensin sodium, Elanco Animal Health, Division of Eli Lilly and Company, Indianapolis, IN 46285, USA). The total mixed ration blends were mixed for five minutes before delivery to each pen replicate using a Reel Auggie Model 3300 feedlot mix wagon equipped with a Digi Star RD-2000-V (4-load cells) electronic scale (Knight Manufacturing Corporation, Brodhead, Wisconsin 53520; Digi Star, Fort Atkinson, Wisconsin 53538). Individual steer final weight within treatment was calculated from each individual hot carcass weight (HCW) and the treatment group dressing percent (DP) (Ʃ T_HCW_/ƩT_LW_ = FT_DP_; HCWi/T_DP_ = FW) where ƩT_HCW_ is treatment HCW sum, ƩT_LW_ is treatment group live weight sum, FT_DP_ is treatment group dressing percent, HCWi is individual steer HCW, and FW is the individual steer calculated final weight.

The GRZ steers grazed perennial NR consisting of cool-season (prairie junegrass—*Koeleria macrantha*, green needlegrass—*Nassella viridula*, bluebunch wheatgrass—*Pseudorognerta spiacata*, slender wheatgrass—*Elymus trachycaulus*, western wheatgrass—*Pascopynum smithi*) and warm-season (blue grama—*Bouteloua gracilis*, switchgrass—*Panicum virgatum*, sideoats *grama—Bouteloua curtipendula*, little bluestem—*Schizachyrium scoparium*, indiangrass—*Sorghastrum nutans*, prairie sandreed—*Calamovilfa longifolia*) grasses from the first week of May to mid-August (108 days). Native range steer grazing equivalents for SF and LF steers were computed from [15] for a standard reference animal being a 454 kg cow grazing up to a six-month-old calf. Converting reference cow weight and SF and LF steer weights to metabolic weight grazing equivalents were 0.840 and 0.934 for SF and LF steers, respectively. Since there was a uniform number of eight steers allotted across treatments and replications, the stocking rate for the LF steers was considered to be ideal and for the SF steers, it was considered to be understocked. Forage availability was adequate all years of the study for LF and SF steers. After NR grazing, the GRZ steers grazed ANN forages starting with field pea-barley intercrop; *Pisum sativum*, var. *Arvika* and *Hordeum vulgare*, var. Stockford) that was followed by grazing UCN that was grown each year within an integrated system consisting of a 5-year, multicrop rotation of crops grown in the semi-arid region of North Dakota. The crop rotation sequence began with hard red spring wheat (T. *aestivum*), which was followed by a multispecies cover crop, UCN, field pea-barley, and sunflower. There were three 1.74 ha ANN forage fields per replicate and each pen replicate stocking rate was 0.2138 ha/steer. Grazing of the field pea-barley intercrop mix was the first ANN forage to be grazed and grazing started when the forage barley was in the immature early-milk stage and peas were 2–3 mm in diameter. The field pea-barley grazing was terminated after an average 32 days of grazing, when pea plants were consumed and barley stubble height was 20.3–24.5 cm. Unharvested corn grazing followed field pea-barley. Unharvested corn grazing was terminated after an average 71 days of grazing, when the higher quality corn aerial plant parts were consumed and predominately stalks remained. After a combined perennial NR and ANN grazing period totaling 212 days, GRZ treatment steers were transferred by commercial truck to the Sustainable Agriculture Research Extension Center feedlot, Lingle, Wyoming, USA, for finishing using the same protocol described for the feedlot control groups.

### 2.5. Forage Nutrient Analysis

Forage nutrient analysis was conducted from samples collected prior to the start of grazing and after grazing ended along individual field transects. The sampling sites were identified and marked for relocation using a handheld Global Positioning System (Garmin, Olathe, KS 66061) and also ground marked with surveyor’s markers. Composited DM forage nutrient analysis for NR, field pea-barley, and UCN are shown in Table 2 and include CP (Kjeldahl procedure), ADF and NDF [16], calcium, phosphorus, ether extract [17], in vitro dry matter digestibility (IVDMD), in vitro organic matter digestibility (IVOMD), [18], and total digestible nutrients (TDN) (81.38 + (CP% × 0.36) − (ADF% × 0.77).

### 2.6. Marketing and Economics

Marketing end point was based on shrunk weight, ultrasound (Aloka SSD-500V; 3.5 MHz-17cm transducer and standoff) fat depth, marbling score, ribeye area (REA) and order buyer confirmation (Cargill Meat Solutions, Ft. Morgan, Colorado 80701). The steers were purchased on a carcass grid basis (Angus America grid). Carcass characteristic measurements included HCW, dressing percent (DP), ribeye area (REA) between the 12th and 13th rib, marbling score (MS), percent Choice quality grade, Warner-Bratzler Shear Force (WBSF), and percent cooking loss.

Native range grazing cost determination (Table 3) was based on a constant cost per kg of body weight (USD 0.002579) and starting BW, end BW, and one-half of the total days grazed to arrive at an annual grazing cost, i.e., (0.002579 × start BW × (total days grazed/2) + (0.002579 × end BW × (total days grazed/2). The mean constant cost per kg of body weight fluctuated during the study period (2013–2015) due to rising beef cattle calf price for 272–318 kg steer calves that exceeded USD 180/45.4 kg [19].

Economic analysis, for the integrated systems, was based on North Dakota Farm and Ranch Business Management Education Program (FBMP), crop enterprise budgets (Region 4) [20]. Annual forage farming enterprise budgets were prepared using actual expenses for seed, fertilizer, chemical, inoculation, and crop insurance (Table 4), which were then integrated with all other expenses in the FBMP, Region 4, database.

Budgets included annual cow cost, backgrounding and grazing cost, and end of grazing steer value estimate (Stockmen’s Livestock Exchange, Dickinson, ND, USA) formed the basis to arrive at net return per steer and net return per ha value at the end of grazing. The North Dakota Farm and Ranch Business Management Education Program annual cow cost budget for Aberdeen/Lowline crossbred cows (mean weight: 522 kg) was adjusted 20.0% to account for small size cow based on cow metabolic weight [5,6,21]. For the finishing budget, steer value at the end of grazing was the feedlot entry cost.

### 2.7. Statistical Analysis

Data was analyzed using Proc MIXED in SAS (SAS Inst. Inc., Cary, NC, USA) [22]. System treatment was a fixed effect and pasture or pen were the experimental unit and basis for random effect. Least-square means were utilized to identify levels of the effects and to control family-wise error adjusted with Tukey. Means were determined to be statistically significant using an alpha level of (*p* < 0.05).

## 3. Results

### 3.1. Grazing Period and Performance

The steers in the study were grown as a common group for modest daily gain (0.60 kg) during the wintering period from November weaning until turnout on NR pastures the first week of May. Table 5 summarizes steer performance during the winter growing period, NR, field pea-barley, UCN grazing, and for the total 212-day grazing period before feedlot entry. Additionally, Table 5 shows the grazing costs for NR, field pea-barley, UCN, protein supplement, and grazing cost per kg of gain.

By design, the grazing steers grazed a sequence of forages starting with perennial native range until the field pea-barley annual forage mix was of a suitable height and condition for grazing to begin, which meant that the field pea-barley was in the milk stage and the peas in pods were small and immature as indicated by the forage CP and ADF when grazing started (CP: 13.26%; ADF: 34.9%). The advancing crop maturity of the field pea-barley, from grazing initiation until approximately 35.0% of the above ground biomass remained, required thirty-two grazing days and from the UCN grazing initiation (CP: 7.73%; ADF: 29.5%) until the higher quality aerial parts were adequately consumed required seventy-one grazing days. During the pre-grazing drylot wintering period, the steer growth did not differ between the treatments (*p* = 0.629). For the 108.3 day NR grazing period, LF steers were 14.1% heavier than the SF steers. However, the grazing growth performance when the steers grazed the annual forage, field pea-barley (*p* = 0.496) and UCN (*p* = 0.776), there was no difference between the two steer frame score types. Considering growth overall for the 212 days, the SF steer ending weight was 11.8% lighter (*p* ≤ 0.001), gain was 10.2% less (*p* = 0.009), and daily growth rate was 10.3% slower (*p* = 0.003). Although grazing steers in the SF category grew more slowly, the grazing cost for NR and ANN forages (field pea-barley and UCN) was less, resulting in lower grazing cost per steer and lower grazing cost per kg of gain (7.80%), indicating that the genetic characteristics of the smaller frame size of the crossbred Aberdeen Angus (Lowline) dams carried over to the smaller framed steer progeny.

### 3.2. Feedlot Performance, Efficiency, and Economics

Feedlot growing and finishing growth, intake, efficiency, and finishing economics are shown in Table 6.

Delaying feedlot entry by grazing the forage sequence for 212 days resulted in an abbreviated 82-day finishing period for the LF and SF grazing steers compared to 218 days for the traditionally finished LF and SF steers (*p* ≤ 0.01), i.e., feedlot finishing days on feed were reduced by 62.4%. Entering the feedlot after extended grazing, LF and SF GRZ steers were an average 62.1% heavier than when FLT control steers entered the feedlot (*p* ≤ 0.01). Looking at SF steers specifically between control and grazing treatments, starting weights were lighter (*p* ≤ 0.01) compared to the LF FLT control and GRZ treatment steers. The small-frame GRZ steer ending weight was 6.73% greater than SF FLT control steer ending weight (*p* ≤ 0.01) and the relationship was similar for the LF GRZ steers that were 6.16% heavier than the FLT control LF steers (*p* ≤ 0.01). Steer growth during grazing did not attain the steer’s full genetic potential for gain; however, the compensating gain in the feedlot was greatest for LF grazing steers followed by SF grazing steers (*p* ≤ 0.01). The compensating grazing steer gain of LF and SF steers was greater than that of the LF and SF feedlot control steers. Naturally, the grazing steers that were on feed 136 less days consumed significantly less total feed per steer (*p* ≤ 0.01); however, daily feed intake (*p* = 0.13) and gain to feed (G:F) efficiency (*p* = 0.59) did not differ between the grazing and control treatments. Although there was no difference identified for daily feed intake per steer and G:F efficiency, feed consumed and feed cost per steer was vastly different (*p* ≤ 0.01). The LF grazing steer feed cost was reduced by 175.9% and the SF grazing steer feed cost was reduced by 165.3%. When other feedlot expenses for yardage, brand inspection, and hospital costs were added to the feed cost and expressed on a cost per kg of gain basis, the LF and SF grazing steer cost was predictably lower compared to the LF and SF feedlot control counterparts (*p* ≤ 0.01).

### 3.3. Carcass Measurement and Meat Quality

Results for carcass trait measurements are shown in Table 7.

Hot carcass weight after a 48 h chill was greatest for LF GRZ steers and the SF feedlot steer carcasses were 20.98% lighter (*p* = 0.01). The LF FLT steers had the second heaviest carcasses weighing 6.39% less than the LF grazing steers and the SF grazing steers were 13.22% lighter than the LF grazing steers (*p* = 0.01). Dressing percent (*p* = 0.01) and marbling score (*p* = 0.02) were greater for SF feedlot and grazing steers; however, although there were numerical differences favoring the SF FLT and GRZ steer carcass, quality grade did not differ (*p* = 0.11). Meat tenderness evaluation using Warner−Bratzler shear force (kg-force) determination did not identify tenderness differences between extended grazing of the NR, field pea-barley, and UCN sequence (*p* = 0.48) and there were no cooking loss differences identified (*p* = 0.43).

### 3.4. Vertically Integrated System Economics

Economic analysis using a vertically integrated business model from birth to slaughter is shown in Table 8.

The table is separated into two parts, i.e., from birth (annual cow cost) to the end of grazing and the feedlot finishing phase through final closeout resulting in the system’s net return value per steer assuming annual SF size cow expenses and SF steer winter background costs were reduced 20%. Therefore, combining annual cow cost, winter backgrounding expense, and grazing expense, the end of grazing net return per SF steer was calculated to be 29.2% more than the LF steers. When the yearling steer system’s net return margin was expressed on a net return per ha basis, the SF steer net return per ha was 41.03% greater than the LF GRZ steers. Steer cost entering the feedlot was less for the SF and LF control steers. Grazing costs combined with the winter backgrounding cost increased the feedlot entry cost for the LF and SF GRZ steers; however, the finishing feed cost for the LF and SF FLT control steers was greater (*p* ≤ 0.01). Hot carcass weights were greater for the LF GRZ steer (*p* = 0.01) and subsequently those carcasses had the highest value; however, carcass value did not differ between treatments (*p* = 0.79). Although no statistical difference was measured for carcass value, inputs relating to the system’s net return favored steers that grazed for an extended period of time before feedlot entry. Compared to the LF GRZ steer net return, the SF GRZ steer, LF FLT steer, and SF FLT steer net returns were −2.32, −36.69 and −42.98% less, respectively, which illustrates the effect managing inputs before feedlot entry has on net return.

## 4. Discussion

### 4.1. Cool- and Warm-Season Annual Forage Management

Annual forages grown in diverse crop rotations present a unique opportunity for cattlemen that manage farm enterprises consisting of both cropland and perennial grass pastures. Previous research identified the reduced risk potential from retaining ownership of yearling steers for grazing, because the inputs are more easily controlled, providing greater opportunity for enterprise profitability [8,9]. A wide array of annual forages that are adapted to both cool- and warm-season environments exist for sequential grazing selection. Annual forages planted on cropland require growing time before grazing can be initiated. Perennial NR provides early spring grazing for livestock while annual forages are being planted and growing until grazing initiation. Based on seeding date and individual crop maturity, sequence grazing starting with perennial native grass forage followed by sequential grazing of annual forages with differing maturity dates afford the grazing manager the opportunity to provide forage that meets the nutrient requirements of the grazing animal for a greater period of time over the entire grazing season. Naturally, forages mature during the grazing period from grazing initiation to the end of grazing. Review of forage maturity change, as shown in Table 2, and steer growth performance shown in Table 5 defines the importance for managing the grazing days of steers for cool-season crops such as the field pea-barley crop grown in this study. The PBLY mixed-crop was placed in the annual forage crop succession as a segue transition forage grown between NR and UCN. Field pea and forage barley are cool-season grain crops bred to mature rapidly for mechanical harvesting; therefore, when planted as grazing crops particular attention must be given to the number of grazing days to insure animal growth performance is not depressed as the forage matures, as was the case in this study. Compared to the NR (108.3 days grazing) that was grazed before PBLY (32.0 days grazing), and UCN that was grazed for 71.3 days after PBLY, steer growth performance grazing PBLY was reduced on average by 43.8%. A probable solution to the reduced steer performance grazing PBLY lies in initiating grazing of PBLY sooner (approximately three days) and reducing the total grazing period length to 27 days. Corn is a versatile crop that can be grown for grazing, chopped for ensiling, or combined for grain production. Grazing corn in western North Dakota is not common and only occurs when the crop is abandoned due to poor growth, during drought, premature frost, or after destruction due to severe hail. Used as a warm-season grazing crop in this investigation, UCN supported yearling steer growth that was comparable to NR and provided 2.33 months of grazing. For the 212-day grazing period, grazing steers grew at approximately 50% of the rate of growth in the feedlot resulting in a compensating growth response, reduced finishing cost, and profitability in the feedlot, which agrees with the results of others [10,11,12].

### 4.2. Cow Size Efficiency

The economics of net return are directly related to managing inputs along the beef supply chain beginning with cow-size management and subsequent management from birth to slaughter. The discussion of cow size has spawned numerous research investigations, but few investigations have studied the relationship between forage resource, extended grazing length, progeny frame size, and system net returns, because biological traits relating to cow−calf production efficiency are not the same as those traits related to a calf’s postweaning growth [23]. Cow efficiency is a complicated and often confusing discussion, because reproduction and feed resources serve an essential role in whether or not a given cow size is efficient within a given environment. Cows that fit into a semi-arid environment are genetically suited to meet maintenance, growth, lactation, and reproduction requirements within the available feed resources [3]. Ritchie [24] described maintenance energy requirements for cows that perform well in reduced forage environments as being cows that have a reduced milk yield, reduced visceral mass, and reduced lean body mass. Whereas, under environments of abundant precipitation and forage availability, high maintenance cows were defined as those with above average milk production, visceral organ weight, and above average lean body mass. Additionally, high maintenance cows do not reach puberty until later in life, compared to SF-size cows [25]. The research results contained herein evaluate the union of cow-size calf generation through postweaning production phases with maximum emphasis on extended grazing prior to feedlot entry as a mechanism combined with cow-size to better manage input costs. Beef cattle are managed in a variety of environments around the globe, are versatile, and fed a variety of diverse feeds adapted to the environment where they are placed [26]. Angus × Hereford crossbred cows have been shown to reduce beef cattle business input costs and maintenance energy needs are similar for younger cows on a forage per unit of BW basis compared to mature cows [27]. Conducted in the semi-arid region of the Northern Great Plains in western North Dakota, years of adequate precipitation are interspersed with periods of drought. Manske [28], reported for June–October growing seasons, during the 119-year period between 1892 and 2010, that 15.1% of the time (18 years) growing seasons were plagued with drought, which requires continuous planning for drought-grazing management. Matching beef cattle to the environment suggests matching cow types to the prevailing feed resources [29]. Based on the work of Miller [30], cow weight impacts feed cost and calf birth weight, which subsequently negatively impact profitability, because feed cost accounts for 63% of annual cow cost. Dickerson [31] stated that the most important aspect contributing to efficiency is reproduction and the ability of a cow to reproduce in the environment in which she is placed is related to cow size, since environmental conditions have the potential to limit economically important genetic traits in beef cattle systems. Therefore, efficient cows are produced when the genetics for growth and milk production match either wet or dry environments, and Arango and Van Vleck [32], when evaluating mature cow weight, determined that profitability maximization is anchored in the forage quality and season of calving. Edwards [33] reinforced the work of [32], determining that attempts to modify the environment with harvested feeds did not improve reproductive efficiency and calf weaning weight was not improved. The USDA Meat Animal Research Center results of a nine-breed comparison of cow types based on feed energy intake reported that cows with genetics for moderate milk production and growth had improved reproductive rates [2]. Therefore, an efficient cow must produce a live calf every year. They categorized the cow types studied into contrasting conditions: (1) cows with genetically greater maintenance requirements, growth, and milk production that were fed lower energy intake compared to (2) cows with genetically lower maintenance, growth, and milk production that were fed higher energy intake. At low energy intake, high maintenance cows could not meet the requirements for milk and growth, and reproduction suffered, whereas low maintenance cows fed high energy intake were unable to convert the additional energy to growth and milk resulting in cows with increase body condition and unchanged calf weaning weight. Selecting cows based on their genetic trait potential for milk and growth is also associated with greater maintenance requirements [2] resulting in greater production costs. Pendell and Herbel [34] and Lalman [35] have suggested that reducing the cost of calf production has greater upside potential for profitability than attempting to increase calf weaning body weight.

### 4.3. Environment, Finishing Performance, and Carcass Measurements

Small improvements in calf weaning weight as well as reduced growth rate performance among progeny from smaller frame-size cows compared to cows that are genetically larger has been reported by others [36,37,38,39] who observed the complexity of cow breed differences, genetics, and environment; and concluded that the combined interaction impacts preweaning calf gain. With the exception of milk production, yearling steer growth in the current study comparing SF and LF steers of differing genetic influences, managed under variable conditions, whether grazing or confined in a feedlot, responded differently within their respective environments and the growth responses for SF cattle can be managed to the producer’s advantage. Stocker cattle grazing performance is highly variable due to forage quality that is influenced by growing season precipitation, soil fertility, and time of season [40,41]. The forage sequence used for this vertically integrated study was designed to provide above average forage quality throughout the grazing season, which resulted in an improved marbling score for the steers that grazed the NR and ANN forage sequence before feedlot entry. This grazing management protocol tends to level out the highs and lows in forage quality over the entire grazing season compared to grazing NR season long in the Northern Great Plains [7]. A slower growth rate due to forage quality characteristics is different from a slower growth rate of steer progeny from smaller sized cows. Although steers from smaller sized cows grew slower during grazing and feedlot finishing, there was no difference in finishing G:F ratio and total finishing cost per kg of gain did not differ between SF and LF steers. Paralleling the slower SF steer rate of gain, lighter HCW for the SF steers was consistent between both FLT and GRZ steer treatments, which is common for progeny from smaller sized cows. However, because the smaller framed cattle reach physiological maturity at an earlier age, the marbling score for both SF FLT and GRZ steers was greater than for the LF FLT and GRZ steers, and SF steer quality grade was numerically improved. Neel et al. [41], evaluated feedlot finishing following stocker cattle winter grazing, and did not identify a difference in finishing performance or carcass characteristics. Subsequently, when DP, marbling score, and quality grade were used to arrive at total carcass value in the current study, there was no difference in carcass value between SF and LF steers from either the FLT or GRZ management methods. Koch [42] and others documented that feedlot grain-finished steers reach Choice quality grade at a younger age than forage-finished steers, but Dinius and Cross [43] documented that cutability for forage-finished steers was higher than grain-fed steers.

### 4.4. Economics and Net Return

Identifying profitable beef production system practices that coordinate brood cow size and postweaning growth, and are compatible with forage resources requires beef cattle enterprise managers to understand the challenges of managing cow size on the one hand and postweaning growth on the other. Research herein shows that smaller sized cows outcrossed to sires with moderate growth potential will produce feeder cattle that grow slower, but perform well in a forage-based retained ownership business model, accompanied by compensating growth during an abbreviated finishing period. In semi-arid production environments, a principle key to profitability is adherence to grazing a sequence of managed forages for 200 days or more, as evidenced by the net return from GRZ LF steers, which compared to the SF GRZ steers was a mere −2.32% less. However, comparing SF FLT steers to SF GRZ steers, SF FLT steers had net return that was −41.63% less pointing to the effect that managed grazing and finishing compensatory gain have on system closeout net return. The SF GRZ steer gain performance was second only to the LF GRZ steer. By contrast, placing SF steers directly into the feedlot compromised growth performance and finishing cost per kg of gain was similar to the LF FLT steers. Coupling the SF FLT steer’s reduced-feedlot performance with reduced carcass value due to light HCW, compared to the other steer management treatments, validates the industry bias and discounts levied against progeny with smaller frame size originating from smaller sized cows. Retained ownership buyers of yearling SF feeder cattle destined for grazing NR or a combination of NR and ANN forages grown on cropland have a buy/sell buyers advantage by purchasing SF stocker cattle at a lower placement cost, but sell on a non-discounted finished fed cattle market.

## 5. Conclusions

Steer calves from small-frame cows grow more slowly than progeny from moderate- to large-frame cows. Feedlot operators responding to a meat packing industry that desires heavier cattle with carcass weights up to 476.2 kg before overweight discounts are applied pay less for small-frame cattle. Environmentally, semi-arid cattle producing regions can very effectively take advantage of the lower production cost and increased pasture-carrying capacity associated with maintaining cows of a smaller frame size that will result in greater net return per ha per cow exposed. Coinciding with smaller frame size cows, to achieve the highest possible net return goal for smaller framed progeny, requires adherence to retained ownership from birth to slaughter utilizing modest winter backgrounding growth, integrated perennial and annual forage sequence managed grazing of 212 days, and a concentrate finishing period that is reduced by 62.4%. A modified integrated management protocol of this type is environmentally sustainable and profitable.

## Figures and Tables

**Table 1:** Beef steer feedlot diet composition and nutrient analysis (DM) ^1^.

Ingredient ^2^	Receiving	Grower	Ration 1	Ration 2	Ration 3	Ration 4	Ration 5
Wheat straw, %	30.0	5.0	5.0	5.0	5.0	3.0	3.0
Alfalfa hay, %	30.0	5.0	5.0	5.0	5.0	3.0	3.0
Corn, whole, %	30.0	40.0	50.0	60.0	70.0	79.5	84.5
Alfalfa haylage, %	10.0	50.0	40.0	30.0	20.0	14.5	9.5
Total, %	100.0	100.0	100.0	100.0	100.0	100.0	100.0
Nutrient analysis							
CP, %	15.8	15.1	13.7	13.8	12.3	12.3	11.3
Fiber, %	18.3	15.8	13.0	12.8	8.9	10.8	6.7
TDN, %	69.6	72.9	76.2	76.5	81.3	78.9	84.0
NEm, MJ/45.4 kg	307.9	327.4	346.7	348.3	376.0	362.2	265.4
NEg, MJ/45.4 kg	193.0	210.2	226.9	228.6	252.5	240.3	63.4
Ca, %	1.44	1.37	1.10	1.02	0.85	0.82	0.66
P, %	0.26	0.30	0.32	0.30	0.31	0.28	0.31

^1^ Senturklu et al., 2018 [7]. ^2^ Feedlot medicated vitamin-mineral supplement. Added to total mixed ration at the rate of 454 gm per steer daily: 12% calcium, 6% phosphorus, 17.5% salt, 2.75% magnesium, cobalt 38.0 ppm, copper 2200.0 ppm, iodine 200.0 ppm, manganese 3300.0 ppm, selenium 35.0 ppm, zinc, 7500.0 ppm, vitamin A 250,000 IU/454 gm, vitamin D 25,000 IU/454 gm, vitamin E 250 IU/454 gm, monensin sodium 250 mg/454 gm.

**Table 2:** Native range and annual forage start and end grazing nutrient analysis (DM).

Forage/Item	CP	NDF	ADF	EE	IVDMD	IVOMD	Ca	P	TDN
Native range									
Start graze	11.02	54.95	30.18	1.97	69.60	68.49	0.50	0.23	59.69
End graze	8.23	66.99	37.91	1.27	54.80	54.05	0.37	0.25	53.50
Pea-barley									
Start graze	9.67	64.67	35.39	1.60	57.46	58.73	0.27	0.13	55.54
End graze	6.94	68.78	38.98	1.97	47.40	48.60	0.31	0.11	52.62
Unharvested corn									
Start graze	7.73	56.64	29.47	1.57	77.95	77.58	0.32	0.24	60.14
End graze	4.55	69.15	38.20	0.66	64.73	63.63	0.17	0.20	53.15

Abbreviations: IVDMD = in vitro dry matter digestibility; IVOMD = in vitro organic matter digestibility; EE = ether extract; Ca = calcium; P = phosphorus; TDN = total digestible nutrients (81.38 + (CP% × 0.36) − (ADF% × 0.77).

**Table 3:** Beef steer native range pasture custom grazing rate calculation ^1^.

	Weight (kg)	Grazing Cost/kg (USD)	Cost/Day (USD)	Days	Period Total Cost (USD)	Grazing Cost/Steer/Day (USD)
GRZ SF						
Date in						
May 1	307.81	0.002567	0.79	54	42.84	
Date out						
August 17	412.69	0.002567	1.06	54	57.43	
Pasture cost/steer				108	100.27	0.93
GRZ LF						
Date in						
May 1	353.21	0.002567	0.91	54	49.15	
Date out						
August 17	475.34	0.002567	1.22	54	66.15	
Pasture cost/steer				108	115.30	1.07

^1^ Three-year mean on a per steer per day basis. Abbreviations: FLT = steers moved directly to the feedlot for growing and finishing; GRZ = steers grazed a sequence of native range, field pea-barley, and unharvested corn before transfer to the feedlot at the University of Wyoming. Steers were slaughtered at the Cargill Meat Solutions, Ft. Morgan, Colorado, USA; SF = small frame, LF = large frame.

**Table 4:** Farming input costs for beef steer annual forage grazing ^1,2^.

Item	Pea-Barley	Unharvested Corn
Corn (pioneer P9690R)	-	143.98
Pea-barley (Perfection pea, Haybet barley), ha	112.95	-
Machine depreciation/ha, USD	15.54	14.80
Fertilizer/ha, USD	-	92.87
Fuel and oil/ha	11.88	13.59
Repairs/ha	15.64	16.13
Inoculant/ha, USD	10.70	-
Chemical—pea-barley (Glyphosate, AMS, Helfire, Rifle D)/ha	30.88	-
Chemical—corn (Glyphosate, AMS, Helfire)/ha	-	21.24
Crop insurance/ha, USD	7.95	27.52
Land rent/ha, USD	70.64	88.28
Subtotal, USD	276.17	418.39
Interest, 5.0%, USD	13.81	20.92
Total crop input cost/ha, USD	289.98	439.31
Cost/steer, USD (cost/ha × 1.74 ha fields)/8 steers	63.07	95.55

^1^ Three-year mean crop expenses. ^2^ Seed, fertilizer, chemical, inoculant, and crop insurance are actual 3-year mean costs/ha. Adjustments to machine depreciation, fuel and oil, and repairs reflect harvesting by grazing. All other expenses are the 3-year mean expenses adopted from crop enterprise budgets [20].

**Table 5:** Effect of forage sequence and extended grazing on yearling beef steer grazing performance ^1^.

Item	GRZ (LF)	GRZ (SF)	SEM	*p*-Value Trt
Total number steers	72.0	72.0		
Field replications. forage/year	3.0	3.0		
Steer frame score	5.29	3.77	3.35	<0.001
Pre-graze winter growing (drylot)				
Winter growing days, day	163.9	161.07	1.405	<0.001
Start Wt., kg	257.09	205.33	13.13	<0.001
End Wt., kg	353.91	305.82	18.47	0.029
Gain, kg	96.82	100.50	7.798	0.743
ADG, kg	0.59	0.63	0.048	0.629
Native range (perennial)				
Grazing days, day	108.33	108.33		
Start Wt., kg	352.72	307.35	19.56	0.003
End Wt., kg.	474.99	412.32	20.23	0.005
Gain, kg	122.26	104.97	2.82	<0.001
ADG, kg	1.13	0.97	0.032	0.003
Field pea-barley (annual)				
Grazing days, day	32.0	32.0		
Start Wt., kg	477.83	414.18	18.58	0.001
End Wt., kg	495.89	434.47	17.96	0.006
Gain, kg	18.06	20.29	2.71	0.568
ADG, kg	0.55	0.63	0.08	0.496
Unharvested corn (annual)				
Grazing days, day	71.33	71.33		
Start Wt., lb	502.61	433.40	18.90	0.0032
End Wt., kg	578.18	509.76	20.06	0.0004
Gain, kg	75.57	76.36	5.07	0.9134
ADG, kg	1.06	1.07	0.06	0.7764
Total grazing (perennial and annual)				
Total grazing days, day	211.7	211.7		
Start Wt., kg	352.72	307.35	19.56	0.003
End Wt., kg	578.18	509.76	20.06	<0.001
Gain, kg	225.43	202.41	5.47	0.009
ADG, kg	1.07	0.96	0.022	0.003
Grazing cost				
Native range (108.33 day), USD	115.30	100.24		
Field pea-barley (32.0 day), USD ^2^	63.07	50.39		
Unharvested corn (71.33 day), USD ^2^	95.55	76.35		
CP supplement, 32%, (0.37kg/day), USD	11.18	11.18		
Grazing cost/head, USD	285.10	238.16		
Grazing cost/kg of gain, USD	1.28	1.18		

^1^ Three-year means. Abbreviations: FLT = steers moved directly to the feedlot for growing and finishing; GRZ = steers grazed a sequence of native range, field pea-barley, and unharvested corn before transfer to the feedlot at the University of Wyoming. Steers were slaughtered at the Cargill Meat Solutions, Ft. Morgan, Colorado, USA; SF = small frame, LF = large frame; Trt = treatment; ADG = average daily gain. ^2^ Grazing cost for SF steers reduced by an adjustment of 20.0% based on cow metabolic weight.

**Table 6:** Effect of beef steer frame score and extended grazing on feedlot finishing performance, efficiency, and economics ^1^.

Item	FLT (LF)	FLT (SF)	GRZ (LF)	GRZ (SF)	SEM	*p*-Value Trt
Number of steers	72.0	72.0	72.0	72.0		
Pen replications frame score/year	3.0	3.0	3.0	3.0		
Frame score	5.63 ^a^	3.82 ^b^	5.53 ^a^	3.77 ^b^	0.26	<0.01
Growth performance						
Grazing days	-	-	211.7	211.7		
Feedlot DOF, days	218.0	218.0	82.0	82.0	3.51	<0.01
Start weight, kg	348.2 ^a^	304.6 ^b^	557.9 ^c^	492.7 ^d^	19.28	<0.01
End weight, kg	687.6 ^a^	595.2 ^b^	730.0 ^c^	635.3 ^d^	23.57	<0.01
Gain, kg	339.4 ^a^	290.6 ^b^	172.1 ^c^	142.6 ^d^	7.65	<0.01
ADG, kg	1.56 ^c^	1.33 ^d^	2.10 ^a^	1.74 ^b^	0.054	<0.01
Feed intake and efficiency						
Feed/steer, kg ^2^	2655.0 ^a^	2171.0 ^b^	1082.0 ^c^	933.0^d^	105.5	<0.01
Feed/steer/day, kg ^2^	12.18	9.96	13.20	11.37	0.447	0.13
G:F, kg	0.1280	0.1335	0.1591	0.1530	0.007	0.59
Finishing economics						
Feed cost/steer, USD	603.74 ^a^	501.87 ^b^	218.85 ^c^	189.20 ^d^	11.42	<0.01
Feed cost/kg gain, USD	1.78 ^a^	1.73 ^a^	1.27 ^b^	1.35 ^b^	0.045	<0.01
Feed, yardage, brand, and hospital cost/steer, USD	674.98 ^a^	572.84 ^b^	247.56 ^c^	218.05 ^d^	11.71	<0.01
Feed, yardage, brand, and hospital cost/kg gain, USD	1.99 ^a^	1.97 ^a^	1.44 ^b^	1.53 ^b^	0.049	<0.01

^a–d^ Means with different superscripts within a line are significantly different, (*p* ≤ 0.05). ^1^ Three-year mean. Abbreviations: FLT = steers moved directly to the feedlot for growing and finishing; GRZ = steers grazed a sequence of native range, field pea-barley, and unharvested corn before transfer to the feedlot at the University of Wyoming. Steers were slaughtered at the Cargill Meat Solutions, Ft. Morgan, Colorado, USA; SF = small frame, LF = large frame; Trt = treatment; G:F = gain:feed; ADG = average daily gain. ^2^ Feed: dry matter basis.

**Table 7:** Effect of beef steer frame score and extended grazing on carcass trait measurements and value ^1^.

Item	FLT (LF)	FLT (SF)	GRZ (LF)	GRZ (SF)	SEM	*p*-Value Trt
Carcass traits						
HCW, kg	397.57 ^c^	349.61 ^d^	422.98 ^a^	373.59 ^b^	13.44	0.01
DP, %	60.22 ^a^	61.09 ^b^	60.19 ^a^	60.84 ^b^	0.21	<0.01
REA, cm^2^	84.69 ^a^	77.08 ^b^	89.85 ^c^	83.85 ^a^	1.59	0.01
MS	611.97 ^a^	640.68 ^b^	583.44 ^c^	631.36 ^a,b^	10.21	0.02
QG, %	93.06	94.24	91.67	97.22	2.73	0.11
Carcass value/steer, USD	2042.47	1753.88	2243.61	2017.51	91.81	0.79
Meat quality						
WBSF, kg-force	2.43	2.42	3.43	2.64	0.06	0.48
Cooking loss, %	17.85	17.61	17.50	15.40	1.17	0.43

^a–d^ Means with different superscripts within a line are significantly different, (*p* ≤ 0.05). ^1^ Three-year means. Abbreviations: FLT: steers moved directly to the feedlot for growing and finishing; GRZ: steers grazed a sequence of native range, field pea-barley, and unharvested corn before transfer to the feedlot at the University of Wyoming. Steers were slaughtered at the Cargill Meat Solutions, Ft. Morgan, Colorado, USA; SF = small frame, LF = large frame; Trt = treatment; MS = marbling score; QG = quality grade; WBSF = Warner−Bratzler shear force.

**Table 8:** Effect of beef steer frame score, extended grazing and retained ownership vertical integration on system net return at the end of grazing and at feedlot closeout ^1^.

Item	FLT (LF)	FLT (SF)	GRZ (LF)	GRZ (SF)	SEM	*p*-Value Trt
Cow−calf and wintering cost						
Annual cow cost, USD ^2^	602.19	508.14	602.19	508.14		
Winter growing cost, USD ^3^	153.32	122.50	153.32	122.50		
Total cost, USD	755.51	630.64	755.51	630.64		
Grazing cost:						
Grazing cost, USD Steer, USD ^4^			285.16	238.11		
Total expense, USD			1040.67	868.75		
End grazing Steer value, USD			1570.45	1553.35	7.37	0.01
Net return/steer, USD			529.78	684.60		
Net return/ha, USD ^5^			26.03	36.71		
Feedlot closeout expenses						
Steer cost, USD	755.51	630.64	1040.67	868.75		
Feedlot cost/steer, USD	674.98 ^a^	572.84 ^b^	247.56 ^c^	218.05 ^d^	11.71	<0.01
Transportation to abattoir, USD ^6^	22.25	19.26	23.86	20.76		
System expense/steer, USD	1452.74	1222.74	1312.09	1107.56		
Income						
Carcass value/steer, USD ^6^	2042.47	1753.88	2243.61	2017.51	91.81	0.79
System net return/steer, USD	589.73	531.14	931.52	909.95		

^a–d^ Means with different superscripts within a line are significantly different, (*p* ≤ 0.05). ^1^ Three-year mean. Abbreviations: FLT = steers moved directly to the feedlot for growing and finishing; and GRZ = steers grazed a sequence of native range, field pea-barley, and unharvested corn before transfer to the feedlot at the University of Wyoming; SF = small frame, LF = large frame; Trt = treatment. ^2^ Expenses are adopted from Beef Cow−Calf Enterprise Analysis and annual cow cost for SF steers adjusted for a 20% carrying capacity enhancement based on cow metabolic weight [5,6,21] of Region 4 values [20]. ^3^ Expenses are the 3-year means adopted from Beef Backgrounding Enterprise Analysis [20]. ^4^ Grazing cost per steer carried forward from Table 4. ^5^ Net return/ha based on sum of native range and annual forage acres grazed per steer. ^6^ Transportation expense per steer from the University of Wyoming, Lingle, WY, USA feedlot that were slaughtered and marketed at Cargill Meat Solutions, Ft. Morgan, Colorado, USA.

## Data Availability

Restrictions apply to the availability of these data by North Dakota State University. Specific data requests are to be directed to Douglas Landblom (douglas.landblom@ndsu.edu).

## Abbreviations

ADF	Acid detergent fiber
ADG	Average daily gain
ANN	Annual forage
DM	Dry matter
DOF	Days on feed
CP	Crude protein
DREC	Dickinson Research Extension Center
DP	Dressing percent
EE	Ether extract
EPD	Expected progeny difference
FBMP	North Dakota Farm and Ranch Business Management Education Program
FLT	Feedlot control treatment
G:F	Gain to feed ratio
GRZ	Grazing treatment
HCW	Hot carcass weight
LF	Large frame
IVDMD	In vitro dry matter disappearance
IVOMD	In vitro organic matter disappearance
LMIC	Livestock market information center
MS	Marbling score
NDF	Neutral detergent fiber
NE	Net energy
NGP	Northern Great Plains
NR	Native range
PBY	Pea barley
QG	Quality grade
UNC	Unharvested corn
REA	Ribeye area
SAREC	Sustainable Agricultural Research Extension Center
SF	Small frame
TDN	Total digestible nutrients
TMR	Total mixed ration
USDA	United States Department of Agriculture
UWF	University of Wyoming feedlot
WBSF	Warner−Bratzler shear force
